# Detection and Monitoring of Insecticide Resistance Mutations in *Anopheles gambiae*: Individual vs. Pooled Specimens

**DOI:** 10.3390/genes9100479

**Published:** 2018-10-03

**Authors:** Konstantinos Mavridis, Nadja Wipf, Pie Müller, Mohamed M. Traoré, Gunter Muller, John Vontas

**Affiliations:** 1 Institute of Molecular Biology and Biotechnology, Foundation for Research and Technology-Hellas, 70013 Heraklion, Greece; 2 Department of Epidemiology and Public Health, Swiss Tropical and Public Health Institute, Socinstrasse 57, 4002 Basel, Switzerland; nadja.wipf@swisstph.ch (N.W.); pie.mueller@swisstph.ch (P.M.); 3 University of Basel, Petersplatz 1, 4003 Basel, Switzerland; 4 Malaria Research and Training Center, University of Science Technique and Technology of Bamako, BP, Bamako 1805, Mali; mohamedmoumine@gmail.com (M.M.T.); GunterCMuller@hotmail.com (G.M.); 5 Pesticide Science Laboratory, Department of Crop Science, Agricultural University of Athens, 11855 Athens, Greece

**Keywords:** vector monitoring, *kdr*, L1014F, L1014S, N1575Y, molecular diagnostics, TaqMan assays, SNPs, pooled samples, insecticide resistant management

## Abstract

Bioassays and molecular diagnostics are routinely used for the monitoring of malaria vector populations to support insecticide resistance management (IRM), guiding operational decisions on which insecticides ought to be used for effective vector control. Previously developed TaqMan assays were optimised to distinguish the wild-type L1014 from the knockdown resistance (*kdr*) point mutations 1014F and 1014S (triplex reaction), and the N1575 wild-type from the point mutation 1575Y (duplex reaction). Subsequently, artificial pools of *Anopheles gambiae (An. gambiae)* specimens with known genotypes of L1014F, L1014S, and N1575Y were created, nucleic acids were extracted, and *kdr* mutations were detected. These data were then used to define a linear regression model that predicts the allelic frequency within a pool of mosquitoes as a function of the measured ΔCt values (Ct mutant − Ct wild type probe). Polynomial regression models showed *r*^2^ values of >0.99 (*p* < 0.05). The method was validated with populations of variable allelic frequencies, and found to be precise (1.66–2.99%), accurate (3.3–5.9%), and able to detect a single heterozygous mosquito mixed with 9 wild type individuals in a pool of 10. Its pilot application in field-caught samples showed minimal differences from individual genotyping (0.36–4.0%). It allowed the first detection of the super-*kdr* mutation N1575Y in *An. gambiae* from Mali. Using pools instead of individuals allows for more efficient resistance allele screening, facilitating IRM.

## 1. Introduction

Since 2000, malaria cases have halved, mostly thanks to case management and vector control interventions, which is estimated to have saved 660 million lives [1]. Around 80% of the disease reduction is attributable to the use of insecticides; however, this success is threatened by increasing insecticide resistance in the malaria mosquito vector. Some mosquito populations show resistance to pyrethroids and other insecticide classes; the prevalence and, therefore, the impact of this phenomenon is increasing every year [2,3,4]. Studies on the molecular mechanisms elucidated target-site and metabolic resistance mechanisms which are associated with pyrethroid resistance in *Anopheles gambiae (An. gambiae)* mosquitoes [5], but also mechanisms including alterations in the insect cuticle that contribute additionally to the emergence of striking multiple resistance phenotypes [6].

Probably the best characterised mechanism of resistance is the so-called knockdown resistance *(kdr)* to pyrethroids and dichlorodiphenyltrichloroethane (DDT). Knockdown resistance arises by point mutations (e.g., L1014F, L1014S) of the target site, the *para*-type sodium channel, leading to reduced insecticide sensitivity [7,8]. An additional *kdr* mutation (N1575Y) has been discovered more recently. The N1575Y mutation enhances the resistance phenotype even further when in combination with the L1014F mutation [9]. Convergent results have identified cytochrome P450 enzymes of the CYP6 subfamily as major contributors to metabolic resistance [10,11]. Some of these genes confer resistance across multiple insecticides [12], but few DNA markers are available [13].

Strategies for insecticide resistance management (IRM) in the context of Integrated Vector Management (IVM) must be evidence-based [14]. Contemporary data on resistance to insecticides is a prerequisite for the implementation of effective interventions. For this purpose, a suite of bioassays (diagnostic dose as well as intensity bioassays and use of synergists) and complementary assays for known molecular markers have been developed to facilitate the implementation of vector control interventions [13].

Bioassays may reliably detect resistance phenotypes at the population level regardless of the underlying cause of resistance, but they require high numbers of live insects, and are not very sensitive to low frequencies of resistance alleles or recessive mutations. Molecular assays complement bioassays in that they may detect resistant alleles at low frequencies at an early stage of resistance development (i.e., incipient resistance) when the effect on the phenotype is not yet apparent. Moreover, molecular assays provide information on the underlying mechanism of insecticide resistance. The molecular assays are applied either on pooled samples in the case of metabolic resistance, with the use of advanced gene expression techniques (Reverse Transcription-quantitative PCR;RT-qPCR, microarrays, RNA-seq), or on individual mosquitoes, with a number of options available; for *kdr* genotyping for example, these include allele-specific PCR, Sequence Specific Oligonucleotide Probe- enzyme-linked immunosorbent assay (SSOP-ELISA), Heated Oligonucleotide Ligation Assay and TaqMan assays (reviewed in [15] and [13]). The latter represents both a reliable (in terms of sensitivity and specificity) and operationally feasible approach (simple one step, closed tube, high throughput, rapid run), and it has been adapted by several labs in malaria endemic countries [13]. However, TaqMan genotyping of individual mosquitoes has a relatively high running cost, as thousands of mosquito specimens are analysed every year worldwide through national vector control programs [16].

However, is it necessary to genotype individual mosquito specimens, or could pooled mosquitoes be used instead to measure allele frequencies for vector control monitoring activities?

The presence and frequency of resistance in mosquito populations (but not individual mosquitoes) is the primary outcome in global databases like the IR Mapper [4], and this data level informs decision making bodies for the appropriate IRM implementation. Examples include the percentage mortality rates which are captured and presented by the bioassay monitoring activities, the levels of detoxification genes which are determined and given as expression levels in pooled mosquito samples, and the frequency of insecticide resistance alleles, irrespectively of whether this latter is captured by analysing individual mosquitoes [4]. Furthermore, in most cases, the frequency of certain insecticide resistance alleles is either absent or close to the fixation levels, which makes the analysis of those traits in individual mosquitoes even more redundant.

Here, we developed and validated a novel approach for monitoring the frequency of pyrethroid resistance mutations in pooled *An. gambiae* samples and discuss its advantages, limitations, and perspectives for application in the framework of IRM.

## 2. Materials and Methods

### 2.1. Mosquito Strains and Field Populations

The *An. gambiae* Kisumu (insecticide susceptible), VK7 (1014F and 1575Y control), and RSP-ST (1014S control) laboratory strains were used for the initial development of the method (Table S1). Field populations originated from Gounkan, Mali, collected during September 2017, were also included in the validation analysis. *Anopheles gambiae* larvae were collected from different breeding sites. Blood fed female *An.gambiae* mosquitoes were collected and put for oviposition in the insectary, while a subset of F1 female adult mosquitoes were collected and stored in RNA*later* (Invitrogen, Carlsbad, CA, USA) until analysis.

### 2.2. Preparation of Pooled Samples

Initially the allelic frequency of the corresponding mutations was determined for the three colonies (Kisumu, VK7, RSP-ST). Artificial pools of 10 adult mosquitoes with known allelic frequency were created. Three replicates for each defined allelic frequency were prepared. To do so, 3–5 days old female mosquitoes (laboratory strains and field populations) were first individually genotyped by extracting genomic DNA (gDNA) from single legs following the DNAzol protocol according to the manufacturer’s instructions, with the following modifications: 200 μL DNAzol reagent (Invitrogen, Cat. No. 10503027), 100 μL of 100% ethanol for DNA precipitation, 10 μL of DEPC treated water for DNA solubilization. Genotyping was performed using the TaqMan assays of Bass et al. [7] for *kdr* point mutations L1014F and L1014S and the Taqman assay of Jones et al. [9] for *kdr* point mutation N1575Y.

### 2.3. Nucleic Acids Extraction from Mosquito Pools

Protocols for nucleic acid (NA) extraction were designed in the framework of the interdisciplinary research project DMC-MALVEC (https://dmc-malvec.eu) with the aim of developing an automated multiplex diagnostic platform (LabDisk) for malaria vectors able to concurrently assess DNA and RNA markers within the same sample [17]. However, the procedure we describe here can also be adapted for conventional DNA extraction protocols.

Mosquito tissue from 10 specimens was mechanically disrupted using a cordless mortar (Sigma-Aldrich, St. Louis, MI, USA, Cat. No. Z359971) and pestle (Sigma-Aldrich, Cat. No. Z359947), with the addition of 200 µL TE buffer (10 mM Tris-HCl, 1 mM EDTA, pH 8.0). Subsequently, NA (DNA and total RNA) was extracted using the MagnaMedics magnetic bead-based protocol with the following modifications to minimise time and materials needed. A total of 150 µL lysis buffer was added to the mechanically disrupted tissue, followed by 10 min incubation at room temperature and a centrifugation step at 16,000 × *g* for 2 min in order to sediment non-lysed tissue debris. The clear lysate obtained was subsequently used to extract NA with the following modifications to the manufacturer’s protocol: 30 µL magnetic beads (MagSi-DNA beads, MagnaMedics, Geleen, The Netherlands, Cat. No. MD01017), 440 µL binding buffer (incubation 10 min in a magnetic stand and removal of supernatant), 200 µL of each wash buffer (incubation 1 min in a magnetic stand and removal of supernatant). Nucleic acid elution was performed with 180 µL elution buffer for 10 min at 50 °C. The integrity of NA was assessed via agarose gel electrophoresis (1.0% *w*/*v*). 

### 2.4. Multiplex Quantitative Polymerase Chain Reaction for Assessing kdr Mutations

Previously published primers and probes were used for the *kdr* 1014F, 1014S [7], and 1575Y [9] assays (Table S2). A triplex assay was optimised for simultaneously detecting the wildtype L1014 and the *kdr* mutations 1014F and 1014S in the same reaction. Each probe was labelled with a different fluorescent dye (HEX for wildtype L1014, FAM for 1014F, and Atto647N for 1014S). The 1575Y assay was used in the duplex format (HEX for wildtype N1575 and FAM for 1575Y), as previously described in Jones et al. [9]. 

All oligonucleotides were optimized using primer/probe matrices for efficiency, sensitivity and specificity (discrimination of wild-type and mutant alleles) in terms of final concentration (Table S2) in the mastermix used in this study, supplied by FTD (Fast-Track Diagnostics, Esch-sur-Alzette, Luxembourg). Total NA of at least 100 ng per sample were used in a total reaction volume of 10 μL. The thermal cycle parameters were: 50 °C for 15 min, 95 °C for 3 min, and 40 cycles of 95 °C for 3 s and 60 °C for 30 s, allowing a qPCR run of approximately 75 min. Samples were amplified in duplicates, and each run always included a non-template control. The analytical parameters of the qPCR reactions are presented in Table S3. Reactions were performed in the ViiA 7 Real-Time PCR System (Applied Biosystems, Waltham, MA, USA). The ΔCt values (Ct mutant probe − Ct wild type probe) were used in each case for the calculation of % allelic frequency in mosquito pools and the development of regression models. Alternative methods of calculating allelic frequency using the *Elongation Factor* (*EF*) gene for normalisation (ΔCt = Ct mutant probe − Ct *EF*) or using solely the Ct values of the mutant probe were also tested. 

### 2.5. Linear Regression Models for Determining Allelic Frequency in Mosquito Pools

The SPSS statistical software was used (SPSS Inc. Released 2008. SPSS Statistics for Windows, Version 17.0. Chicago, IL, USA). A training set of pools of 10 mosquitoes composed of different numbers of individuals from the lab colonies with known *kdr* mutation status was used to fit linear regression models that predict allelic *kdr* frequencies as a function of measured ΔCt values. Both linear and polynomial (quadratic) regression models were fitted and the best model chosen on the basis of adjusted R squared and residual plot analysis (goodness of fit), standard error of estimate (accuracy), and *p* values (significance). An independent validation set of artificial populations was used to corroborate the predictive value of the selected models from the training set, and also to calculate the average margin of error. For this reason, the equations developed were applied to calculate % allelic frequencies from ΔCt values and the average difference between predicted and expected values was calculated for each trait.

Variance due to pooling formation (σ^2^_pf_) was calculated as described in [18] using triplicate mosquito pools of several allelic frequencies for each point mutation, and calculating the median variance in the predicted % allelic frequency. The method’s accuracy and precision were calculated according to Lavebratt et al. [19]. Precision was measured as the within population standard deviation of the difference between estimated and actual allele frequency (D). The mean value of |D| between different populations was used for the calculation of accuracy. Artificial pools having a wide range of allelic frequencies were used for the estimation of accuracy and precision. The limit of detection (LOD) was defined as the lowest allelic frequency that could be reliably detected in a mosquito pool of 10 individuals. *p* values < 0.05 were considered statistically significant.

## 3. Results

### 3.1. Quality Control of quantitative Polymerase Chain Reaction Assays and Genotyping of Mosquito Control Strains

The developed method determines allelic frequencies quantitatively for the triplex L1014F/S and the duplex N1575Y assays. Relevant analytical parameters such as reaction efficiency, linearity, dynamic range, and percent Coefficient of Variation (% CV) for Ct values are presented in Table S3.

The *kdr* mutation status of each strain was initially determined (Table S1). The Kisumu strain was 100% wild type for all mutations, 1014F and 1014S were found with a frequency of 100% in VK7 and RSP-ST, respectively. The 1575Y mutation was found with a 40% frequency in the VK7 strain.

### 3.2. Development of Regression Models with the Training Set

Linear regression models were developed for all three *kdr* point mutations (1014F, 1014S, and 1575Y). In all cases, quadratic models showed superior performance compared to models with a single term in terms of goodness of fit, estimated by adjusted R squared values and residual plot analysis (Figure S1), and in terms of accuracy, estimated by the standard error of the estimate (Table 1).

**Figure 1:**
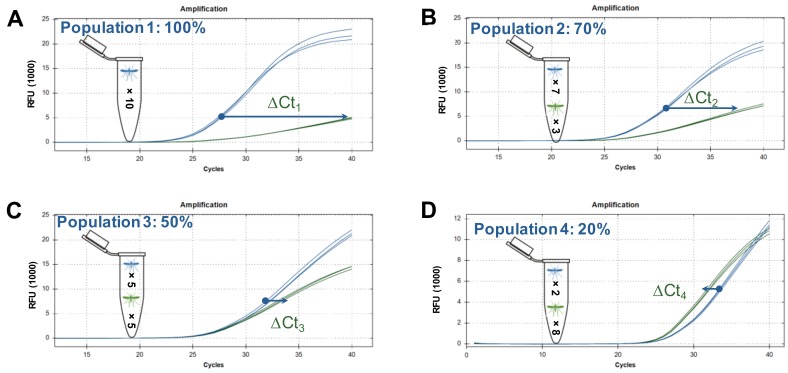

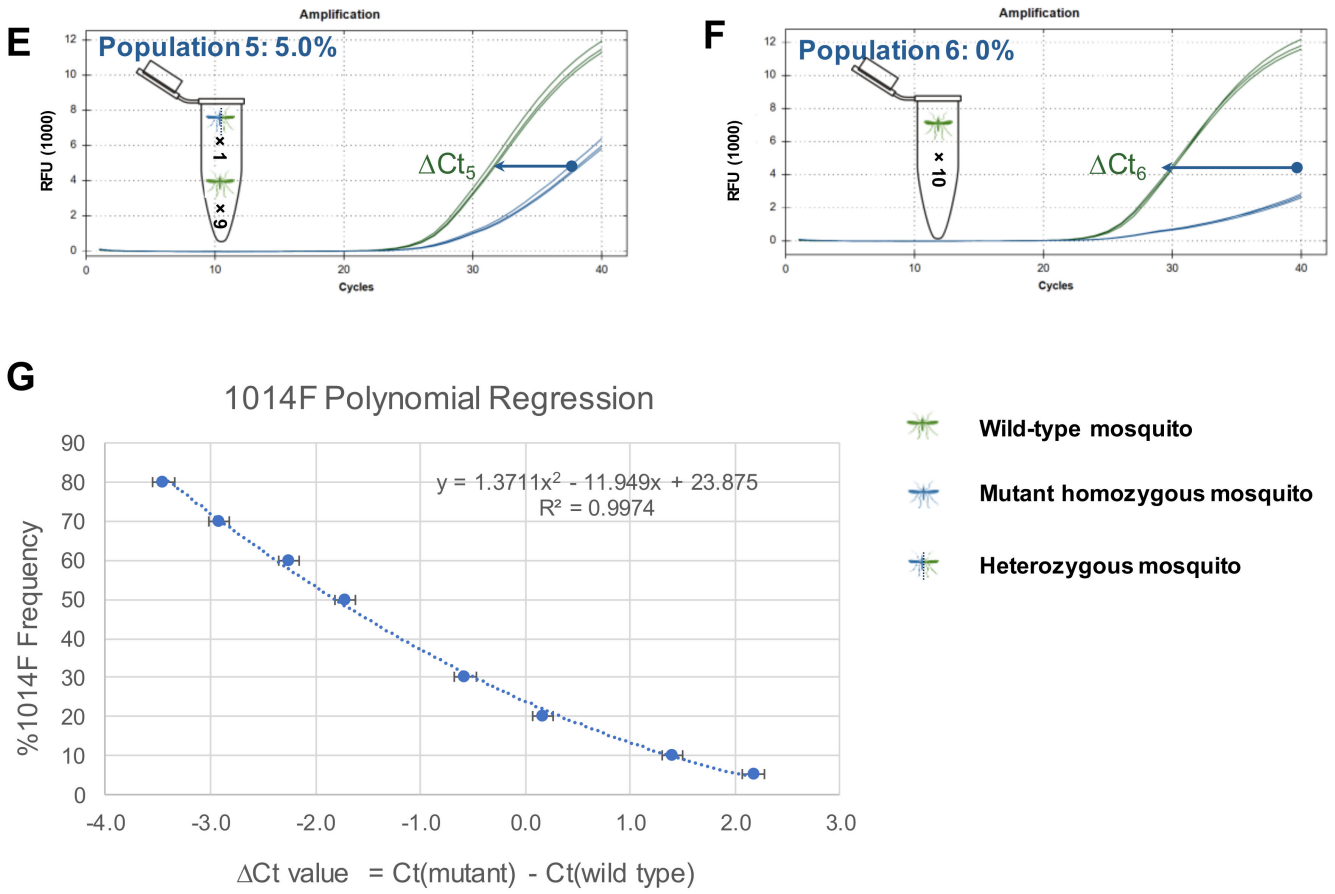
Development of regression models. *A-F*: Reaction curves for L1014F showing the increasing difference between cycling of mutant and wild type probes from higher to lower population frequencies of the mutant allele. *G*: Polynomial regression curve for 1014F.

Alternative methods of estimating allelic frequencies by normalising the signal of the mutant probe with an independent assay (i.e., EF exon), and by using the Ct value of the mutant probe only, were also explored but showed suboptimal results when compared to the ΔCt approach (Table S4). Therefore, polynomial regression models using the ΔCt as the predictive value were selected for further analysis. An example of this approach is presented for the 1014F allele in Figure 1.

The developed models show a good fit for all three mutations with adjusted R squared values in the range of 0.996–0.997 (Table 1), and no signs of dependencies in the residuals (Figure S1). The variance due to pooling formation ranged from 2.2 to 4.9% (Table 1).

All models were able to reach as low as 5.0% in allelic frequency (Table 1) with the training set, translating to a detection limit of one heterozygous mosquito out of a pool of 10; this is the lowest detection rate that could possibly be required for a pool of 10 mosquitoes.

### 3.3. Validation of Regression Models

The developed regression models were applied to calculate allelic frequencies in an independent series of samples with known allelic frequencies for each mutation (validation set). The margin of absolute error in determination (accuracy) between true and estimated allelic frequencies was in the range of 3.26–5.9%, depending on the locus (Table 2). Variability in determinations within populations of the same frequency (precision) was also low (1.66–2.99%) (Table 2).

The correlation coefficient between estimated and true allele frequencies ranged between 0.959 and 0.989 (*p* < 0.001). When looking at individual populations covering different scales of allelic frequencies, the average differences between actual and measured mutant allelic frequency values were 7.7% for high- (60–80%), 3.7% for mid- (20–50%) and 2.1% for low- (5–15%) allelic frequency populations (Table 3).

We also developed specific models to cover the (rather rare) case where populations are comprised exclusively of a mixture of mutant 1014F and 1014S mosquitoes (Table S5). By using the 3-plex L1014F/S assay, these populations can be identified by an undetectable Ct signal for the wild type probe, and a concurrent detectable signal for both mutant probes (1014F, 1014S). As the wild type Ct value cannot be used in such cases, we investigated the development and validation of models using the EF exon assay an independent normaliser, or solely the Ct value of the mutant probe as input for the estimation of allelic frequencies. A new series of artificial populations consisting of VK7 (1014F homozygous mutant) and RSP-ST (1014S homozygous mutant) individuals in various proportions was used following the approach described above. The results obtained from using the Ct value from the mutant probe as input were more consistent compared to those obtained with EF exon as normaliser, and are presented in the Table S5. For 1014F the accuracy was 6.45% and the precision 2.37%, whereas for 1014S the values were 14.27% and 10.31%, respectively.

In order to further validate our approach, we applied it to previously collected *An. gambiae* field populations from Mali (Gounkan area). In a first step, we genotyped the legs of N = 30 individuals to obtain the accurate allele frequency. Next, we genotyped three pools of each 10 randomly chosen remaining bodies to estimate the allele frequency with our new model. Results from individuals showed a 1014F allelic frequency of 75.0% (N = 18 mutant homozygotes, N = 9 heterozygotes, N = 3 wild-type homozygotes) compared to 71.0%, as assessed by the average of the three pools, indicating a difference of only 4.0 % between the two methods. The 1575Y mutation was detected at a frequency of 30.0% in N = 30 individuals (N = 1 mutant homozygotes, N = 16 heterozygotes, N = 13 wild-type homozygotes), compared to 29.64% when analysing the corresponding samples in pools. The difference between the two methods in this case was negligible (equal to 0.36%).

## 4. Discussion

Reducing the time and cost of determinations is essential for vector control monitoring in the frame of IRM applications. Here, we developed and validated a novel approach to reliably calculate allelic frequencies of major *An. gambiae kdr* mutations (L1014F, L1014S, N1575Y) in pooled mosquito samples. Firstly, optimisation of previously developed TaqMan assays now allows the detection of *kdr* 1014F and 1014S and L1014 alleles in a single triplex reaction; this was previously performed in two separate reactions. Secondly, the presence and frequency of resistant alleles was accessed in pooled mosquito samples, and the outcomes were compared with the outcomes derived by “gold standard” *kdr* assessment in individual mosquitoes. The developed method allows the detection of allelic frequencies of as low as 5.0%. This means that a single heterozygous mosquito can be reliably detected in a pool of 10 mosquitoes in which all the remaining individuals are wild type. The method is both precise (Precision = 1.66–2.99%) and accurate (Accuracy = 3.3–5.9%). The variance in pool formation was smaller than 5%, which is reasonable and not unexpected, considering that the natural variability in body size will affect the allele frequency measured per mosquito.

Allelic frequencies measured in field caught samples using individual mosquitoes or pools were very similar; for 1014F, the difference between the two approaches was 4.0%, and for 1575Y, the corresponding difference was 0.36%. Both these figures are practically negligible in the frame of monitoring applications for evidence based IRM decisions. In practice, very often fixation levels or the absence of certain mutations are recorded, and intermediate levels of resistance alleles are rather rare. Moreover, in the pilot application, the super-*kdr* N1575Y mutation was detected for the first time in *An. gambiae* mosquitoes from Mali.

The novel approach we describe here does not distinguish between homozygotes and heterozygotes. This is a limitation, as the operationally relevant pyrethroid resistance phenotype is primarily expressed when mosquitoes are at the homozygote RR stage. However, given that the frequencies commonly detected in the field are often very high or very low, this information might be more relevant in research projects but not for decision making in IRM. Moreover, the outcome of the assay is fully compatible with the widely used IR Mapper database (www.irmapper.com), a tool that currently drives decision making for IRM at various stakeholder levels. Required entry fields in this data base are “test method used”, “mechanism tested”, “number of mosquitoes used per assay”, “outcome of the assays (detected/ not detected)” and “frequency of mutations”. All this information is readily available by using a pool strategy, yet at a substantially reduced cost and time. An approximately 10-fold reduction in cost (i.e., for nucleic acid extraction reagents, qPCR reagents, and other consumables and labour for nucleic acid extractions) for analysing mosquitoes for *kdr* mutations is possible with the new approach, while throughput and analysis time are also substantially reduced.

In principle, our method can be applied to any single nucleotide polymorphism (SNP), with only minimal requirements for optimization and assay development. A quick overview on how to setup the experimental procedure to calculate allelic frequencies from mosquito pools is presented in Figure S2. The rationale of the method we describe here can be also applied to other traits, such as the iAche (G119S) mutation [20] conferring resistance to organophosphate and carbamate insecticides, as well as the monitoring of malaria vector species identification markers [21].

The application of *Plasmodium* detection assays on mosquito pools has been also introduced in many low transmission malaria countries to save time and resources [22]. A novel method with no requirement for dissection and post-PCR processing was recently developed for the detection of *Plasmodium falciparum* infective mosquitoes [23]; with high sensitivity, it can detect infective-stage specific parasite transcripts in samples containing a mix of infective with non-infected mosquitoes at a 1:100 ratio. The feasibility of analysing mosquito pools, maintained in RNA*later* (Invitrogen, ) solutions, sets the basis for the development of more holistic and automated approaches to vector population monitoring [24], as the same biological material e.g. a mosquito pool, can be used both for DNA-markers, such as target site mutations and species identification, as well as for RNA-markers (transcript levels of major detoxification genes, infective-stage plasmodium transcripts). These assays are suitable for integration within multiplex sample-to-answer automated diagnostic platforms, such as the DMC-MALVEC-LabDisk for malaria vectors and the ArboVec-LabDisk for arbovirus vectors that are currently under development (https://dmc-malvec.eu, https://infravec2.eu) [17], and are in line with applications in human diagnostics [25].

## 5. Conclusions

We tested a simple research question: "Can we genotype pools of mosquitoes and still retrieve the same information on point mutations as we would using single individuals?”. Working with the example of *kdr* mutations, we optimised existing TaqMan qPCR assays and developed reliable regression models to analyse data from pooled specimens. The novel approach is able to measure allele frequencies from pools of ten mosquitoes with very high accuracy, i.e., equivalent to genotyping individual mosquitoes, but requiring substantially less time and lower cost. Vector control monitoring activities in the frame of IRM programmes may greatly benefit from the described approach.

## Figures and Tables

**Table 1:** Polynomial regression models for the detection of 1014F, 1014S, and 1575Y in mosquito pools (training set).

*Kdr* Mutation	Equation	R Square (Adjusted)	SE of the Estimate	*p* Value	*σ^2^*_pf_ Mean (Range)	Detection Limit
1014F	%MAF = 1.37 × (ΔCt)^2^ − 11.9 × (ΔCt) + 23.9	0.996	1.72	3.55 × 10^−7^	4.9 (0.72–10.6)	5.0%
1014S	%MAF = 1.11 × (ΔCt)^2^ − 10.6 × (ΔCt) + 21.7	0.996	1.53	1.07 × 10^−4^	2.9 (1.6–12.1)	5.0%
1575Y	%MAF = 5.68 × (ΔCt)^2^ + 35.4 × (ΔCt) + 60.0	0.997	1.28	5.88 × 10^−5^	2.2 (0.13–16.6)	5.0%

MAF: Mutant Allele Frequency; ΔCt = (Ct_mutant probe_ - Ct_wild-type probe_); SE: Standard error; σ*^2^*_pf_: Variance in pool formation.

**Table 2:** Application of the developed regression models in an independent set of artificial populations (validation set). Calculation of accuracy and precision of the method and correlation between true and estimated allelic frequencies.

*kdr*	Accuracy ± SE	Precision (Range)	r_s_	*p* Value
1014F	3.58 ± 0.84	2.99 (1.73–3.66)	0.978	5.20 × 10^−6^
1014S	5.9 ± 1.5	2.32 (0.69–4.1)	0.989	2.50 × 10^−5^
1575Y	3.26 ± 0.62	1.66 (0.517–3.86)	0.959	8.37 × 10^−7^

r_s_: Pearson correlation coefficient between true and estimated allele frequencies.

**Table 3:** Validation of the developed regression models: Differences in actual versus measured allelic frequencies. For populations where N= 2 replicates were analysed, the mean value ± SE of % allelic frequency is given.

*kdr*	Species	Population	Individuals Genotyped	Actual MAF	Measured MAF ± SE	Absolute Difference
1014F	*An. gambiae s.s. S-form* and *An. gambiae s.s. M-form*	P1a, P1b	RR =6; RS = 0; SS = 4	60%	55.54% ± 2.25	4.46%
		P2a, P2b	RR =3; RS = 0; SS = 7	30%	33.14% ± 1.97	3.14%
		P3a, P3b	RR = 2; RS = 0; SS = 6	20%	25.35% ± 2.59	5.35%
		P4a, P4b	RR = 1; RS = 0; SS = 9	10%	11.60% ± 1.22	1.60%
		P5a	RR = 1; RS= 0; SS = 18	5%	1.90%	3.1%
1014S	*An. gambiae s.s. S-form*	P6a, P6b	RR = 8; RS = 0; SS = 2	80%	90.96% ± 1.64	10.96%
		P7a	RR = 5; RS = 0; SS = 5	50%	45.73%	4.27%
		P8a, P8b	RR = 3; RS = 0; SS = 7	30%	33.54% ± 0.49	3.54%
		P9a, P9b	RR = 1; RS = 0; SS = 9	10%	14.00% ± 2.89	4.00%
1575Y	*An. gambiae s.s. S-form* and *An. gambiae s.s. M-form*	P10a	RR = 4; RS = 2; SS = 4	50%	47.94%	2.06%
		P11a, P11b	RR = 1; RS = 4; SS = 5	30%	33.97% ± 1.05	3.97%
		P12a, P12b	RR = 1; RS = 1; SS = 8	15%	13.83% ± 2.19	1.17%
		P13a, P13b	RR = 1; RS = 0; SS = 9	10%	9.63% ± 3.56	0.37%
		P14a, P14b	RR = 0; RS = 1; SS = 9	5%	7.59% ± 1.85	2.59%

MAF: Mutant allele frequency; SE: Standard error; *s.s.:* *sensu stricto*; RR: Mutant homozygotes; RS: Heterozygotes; SS: Wild-type homozygotes.

## Supplementary Materials

The following are available online at http://www.mdpi.com/2073-4425/9/10/479/s1, Table S1: Mosquito specimens suitable for use as artificial populations; Table S2: Primers and probes used in the present study; Table S3: Quality control characteristics of the qPCR reactions; Table S4: Alternative methods of calculation using normalization with exon EF or the Ct value of the mutant probe; Table S5: Results from populations where only 1014F an 1014S individuals were present in different proportions (no wild type mosquitoes present); Figure S1: Residual plot analysis for the selection of optimal regression model between linear and polynomial regression models; Figure S2: A practical approach on how to setup the experimental procedure to calculate allelic frequencies from standard mosquito pools and how to apply it in each run for unknown pooled samples.
