# Supplementary material for: Detection and Monitoring of Insecticide Resistance Mutations in Anopheles gambiae: Individual vs. Pooled Specimens
Source: Genes (Basel). 2018 Oct 3;9(10):479. doi: 10.3390/genes9100479 (PMC6209882; doi:10.3390/genes9100479)
Supplement: Supplementary file 1 [file genes-09-00479-s001.pdf]

Supplementary Table S1. Mosquito specimens suitable for use as artificial populations (standards for the regression models) that are available from Biodefense and Emerging Infections Research Resources Repository (BEI Resources, www.beiresources.org), and Liverpool Insect Testing Establishment (LITE, www.lite-testing-facility.com).

SNP	Strain	Species	Resource	Alternatives
L1014 (wild type)	KISUMU1	*An. gambiae s.s. S-form*	BEI resources	Ngousso, ASEMBO1, Moz
1014F (mutant)	VK7	*An. gambiae s.s. M-form*	LITE	AKRON, TIASSALE
1014S (mutant)	RSP-ST	*An. gambiae s.s. S-form*	BEI resources	RSP
N1575 (wild type)	KISUMU1	*An. gambiae s.s. S-form*	BEI resources	Ngousso, ASEMBO1, Moz
1575Y (mutant)	VK7	*An. gambiae s.s. M-form*	LITE	N/A

Supplementary Table S2. Primers and probes used in the present study.

Oligo.	Assay	Sequence (5′–3′)	Modification	Concentration (nM)
kdr_For	1014F/S	CATTTTTCTTGGCCACTGTAGTGAT	None	500
kdr_Rev	1014F/S	CGATCTTGGTCCATGTTAATTTGCA	None	200
kdr-wt(L)_Pr	1014F/S	CTTACGACTAAATTTC	5′HEX, 3′MGB	500
kdr-mt(F)_Pr	1014F/S	ACGACAAAATTTC	5′FAM, 3′MGB	500
kdr-mt(S)_Pr	1014F/S	ACGACTGAATTTC	5′Atto647N, 3′MGB	500
1575_For	1575Y	TGGATCGCTAGAAATGTTCATGACA	None	500
1575_Rev	1575Y	CGAGGAATTGCCTTTAGAGGTTTCT	None	200
1575-wt(N)_Pr	1575Y	ATTTTTTTCATTGCATTATAGTAC	5′HEX, 3′MGB	300
1575-mt(Y)_Pr	1575Y	TTTTTCATTGCATAATAGTAC	5′FAM, 3′MGB	400
exon_EF_For	EF exon	AGCAGCTGTTCAGCAAAACG	None	100
exon_EF_Rev	EF exon	TCTCCCGCACAGTGAAAGAC	None	200
exon_EF_Pr	EF exon	ACATGCTGATGCCCGGCGATC	5′Atto647N, 3′MGB	300

For: Forward; Rev: Reverse; Pr: Probe; wt: wild type; mt: mutant.

Supplementary Table S3. Quality control characteristics of the qPCR reactions.

Assay	% Efficiency	Linearity (R square)	Dynamic Range (Ct values)	CV
**kdr L1014-F/S (3-plex)**				
Wild type probe (HEX)	99.46	0.998	24.0–33.0	1.3%
1014F mutant probe (FAM)	99.01	0.995	24.0–33.0	1.2%
1014S mutant probe (Atto647N)	94.15	0.999	23.5–33.0	1.5%
**kdr N1575Y (2-plex)**				
Wild type probe (HEX)	100	0.999	25.0–32.0	1.2%
1575Y mutant probe (HEX)	105	0.999	25.0–32.0	1.3%
**Exon EF (single plex)**				
Exon EF probe (Atto647N)	98.03	0.996	22.0–32.0	1.1%

CV: Coefficient of Variation.

Supplementary Table S4. Alternative methods of calculation using normalization with exon EF or the Ct value of the mutant probe.

Kdr L1014F	Exon EF normalization	Mutant probe Ct
Equation	$MAF_{1014F} = 7.2 \times (\Delta Ct)^2 - 41.58 \times (\Delta Ct) + 66.04$	$MAF_{1014F} = 3.3 \times (Ct)^2 - 177 \times (Ct) + 2389$
R square (adjusted)	0.981	0.869
p value	2.2×10^{-5}	0.006
Accuracy of %MAF determination ± SE	7.99 ± 1.92	13.00 ± 4.36
Precision of %MAF determination (Range)	3.75 (0.35-5.58)	7.48 (0.02–17.17)

MAF: Mutant allele frequency; $\Delta Ct = (Ct_{mutant\ probe} - Ct_{exonEF})$

Supplementary Table S5. Results from populations where only 1014F an 1014S individuals were present in different proportions (no wild type mosquitoes present). In this scenario, the probe signal from the wild-type probe cannot be used for normalization since it is non-specific and the undetected range (>33.0). Calculations shown here were performed using the Ct values of the mutant probe.

kdr	1014F	1014S
Equation	$MAF_{1014F} = 2.3 \times (Ct_{mut})^2 - 126 \times (Ct) + 1723$	$MAF_{1014S} = 7.7 \times (Ct_{mut})^2 - 373 \times (Ct_{mut}) + 4547$
R square (adjusted)	0.9813	0.9854
p value	0.019	0.015
σ^2_{pf} mean (range)	17.5 (0.13–25.0)	23.7 (2.2–40)
Accuracy of %MAF determination ± SE	6.45 ± 2.61	14.27 ± 2.35
Precision of %MAF determination (range)	2.37 (0.61–6.15)	10.31 (4.01–13.41)
r_s (*p* value)	0.865 (6×10^{-3})	0.776 (0.024)

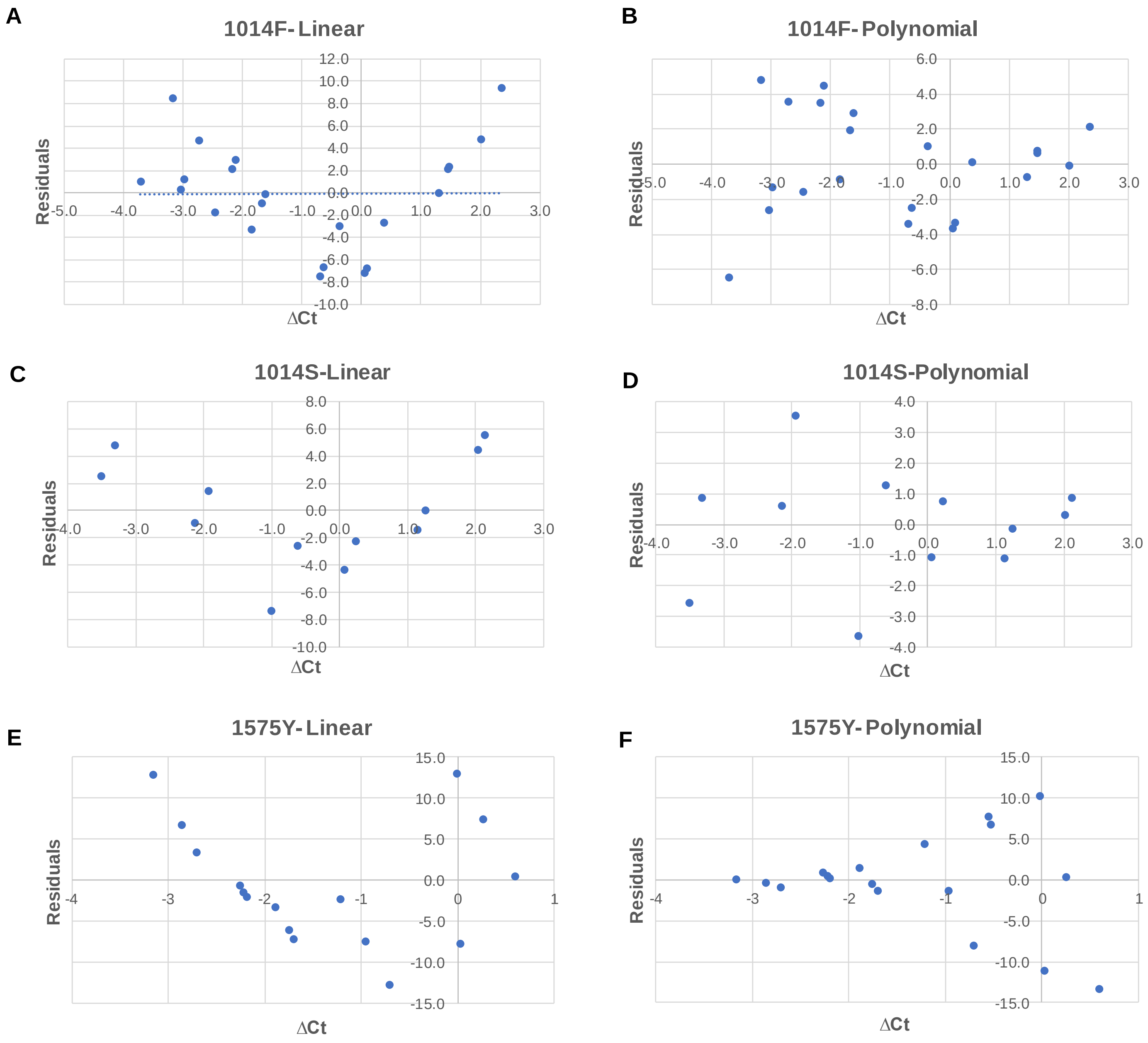

Supplementary Figure S1 Residual plot analysis for the selection of optimal regression model between linear (A, C, E) and polynomial (B, D, F) regression models. Residuals are the difference between the observed and the predicted value of the dependent variable. Residual plots were constructed using the residuals on the vertical axis and the independent variable in the horizontal axis. The rather random scatter of residuals of the polynomial model shows its comparative advantage versus the linear models that show a clear U-shaped scatter.

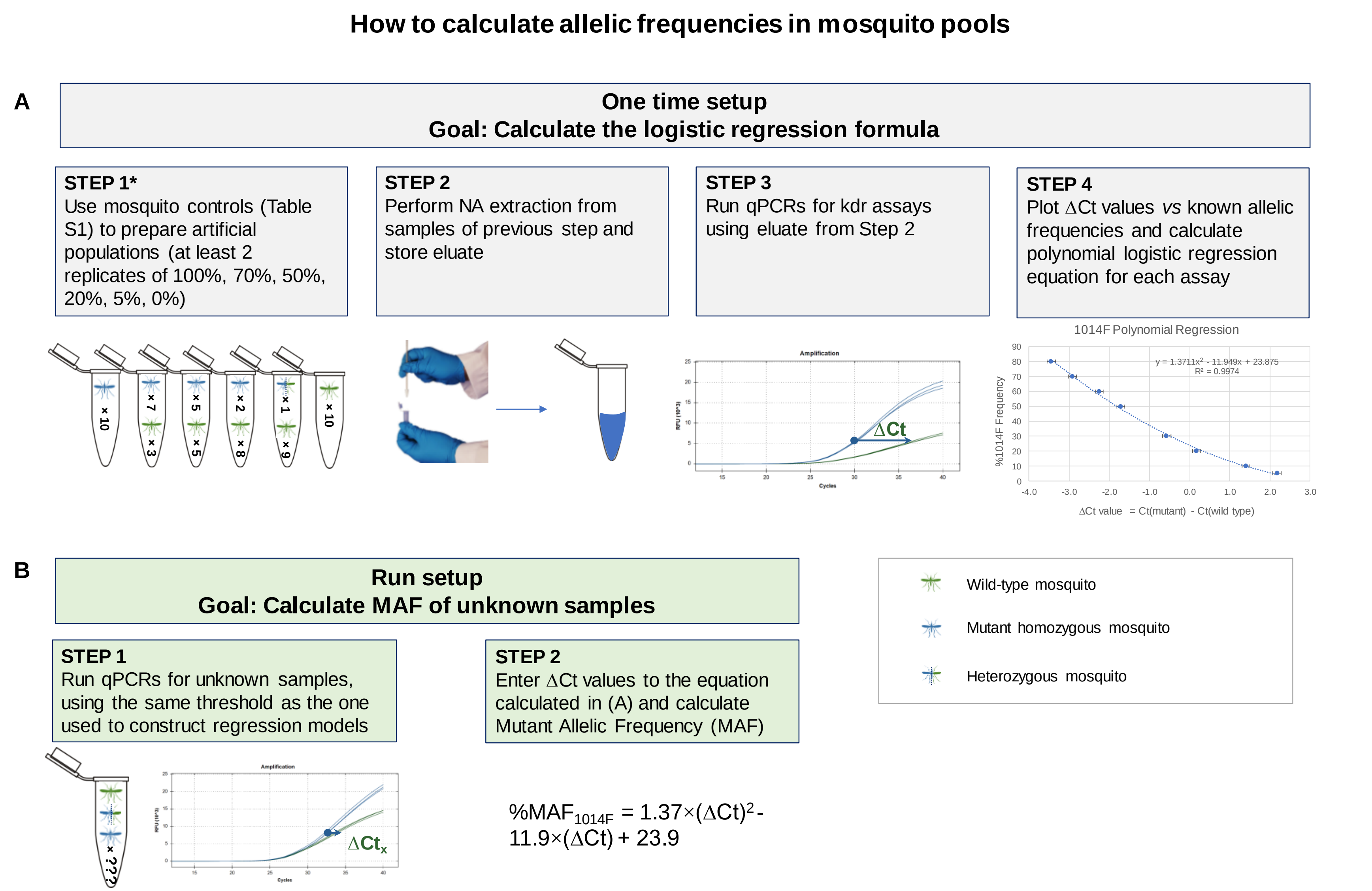

Supplemental Figure S2 A practical approach on how to setup the experimental procedure to calculate allelic frequencies from standard mosquito pools (Part A) and how to apply it in each run for unknown pooled samples (Part B). Stored eluates from part A, step 2 are available upon reasonable request.
